# Preliminary study on the correlation between thyroid magnetic resonance parameters and radiation dose after radiotherapy for nasopharyngeal carcinoma

**DOI:** 10.3389/fpubh.2024.1526147

**Published:** 2025-01-07

**Authors:** Kuan Lu, Chenxia Zhou, Jiaming Ren, Jialu Ni, Weisen Yang, Yeqing Wang, Dan Jin, Jianjun Qian, Yaqun Zhu, Dai Shi, Wentao Hu, Liang Xu

**Affiliations:** ^1^The Second Affiliated Hospital of Soochow University, Suzhou, China; ^2^Suzhou Medical College, Soochow University, Suzhou, China; ^3^State Key Laboratory of Radiation Medicine and Protection, School of Radiation Medicine and Protection, Collaborative Innovation Center of Radiological Medicine of Jiangsu Higher Education Institutions, Soochow University, Suzhou, China

**Keywords:** thyroid, magnetic resonance imaging (MRI), nasopharyngeal carcinoma, radiation therapy, dose

## Abstract

**Background:**

Hypothyroidism is a common sequela after radiotherapy for nasopharyngeal carcinoma (NPC). Magnetic resonance imaging (MRI) has gained prominence in thyroid imaging, leveraging its non-ionizing radiation, high spatial resolution, multiparameter and multidirectional imaging. Few previous studies have investigated the evaluation of radiation-induced thyroid injury by MRI.

**Methods:**

MRI and radiotherapy data of 32 patients who were first diagnosed with nasopharyngeal carcinoma in our hospital from April 2015 to April 2024 and underwent radiotherapy in the radiotherapy department were retrospectively collected. Before, during and after radiotherapy, the thyroid morphology was observed on MR images, and the quantitative parameters of size (width, thickness) were measured on T1-weighted images. The signal intensity (SI) of the thyroid gland was measured on T1-weighted imaging (T1WI), T2-weighted imaging (T2WI) and contrast-enhanced T1-weighted imaging. The differences in thyroid parameters at different time points before and after radiotherapy were compared. The correlation between the MRI quantitative parameters of the thyroid and the radiation dose volume of the thyroid and the radiation dose of the pituitary were analyzed.

**Results:**

The width, thickness and volume of the thyroid decreased gradually before, during and 6 and 12 months after radiotherapy. They were negatively correlated with the mean thyroid dose and V50 (*p* < 0.05), but were not significantly correlated with the maximum and minimum thyroid doses, V30 and V35 (*p* > 0.05). The T1WI relative signal intensity (RSI), T2WI RSI, and enhanced T1WI RSI of the thyroid gland gradually decreased from before radiotherapy to during radiotherapy and 6 months and 12 months after radiotherapy. The T1WI RSI, T2WI RSI, and enhanced T1WI RSI during radiotherapy and 6 months and 12 months after radiotherapy were negatively correlated with the mean radiation dose, V40, V45, and V50 of the thyroid gland (*p* < 0.05), but were not significantly correlated with the maximum radiation dose, minimum radiation dose, V30, and V35 of the thyroid gland or the radiation dose of the pituitary gland (*p* > 0.05).

**Conclusion:**

Quantitative MRI analysis can non-invasively and effectively show the changes in thyroid shape, size and signal intensity in patients with nasopharyngeal carcinoma before and after radiotherapy, which is crucial for early and accurate assessment of thyroid damage, enabling timely treatment to preserve thyroid function.

## Introduction

Nasopharyngeal carcinoma (NPC) is one of the most common malignant tumors in southern China and Southeast Asia; it accounts for 78.29% of head and neck malignant tumors (1). Intensity-modulated radiotherapy (IMRT) has the advantages of high positioning accuracy and radiation accuracy, which can increase the radiation dose for complex tumor targets while reducing the irradiation dose of surrounding normal tissues (2). It has become the first choice of treatment for nasopharyngeal carcinoma (3, 4). However, even in the IMRT era, NPC survivors still face long-term sequelae which negatively affect their quality of life (5). Hypothyroidism (HT), as one of the most common late toxicities, has been reported to occur in 40–50% of patients who were treated with neck irradiation (6–8). Therefore, the prevention and monitoring of radiotherapy-induced hypothyroidism is particularly important in the era of IMRT for nasopharyngeal carcinoma treatment (9).

Laboratory assessment and ultrasonography examination are the main evaluation methods of thyroid function. However, the former is often delayed due to the concealed clinical symptoms and the neglect of doctors and patients, and the disadvantage of the latter is that the accuracy of results is greatly affected by operators. MRI is the preferred routine examination method for patients with nasopharyngeal carcinoma before and after radiotherapy. It has good soft tissue contrast, multiparameter and multidirectional imaging, and the contrast agent is generally gadolinium, which has little effect on thyroid function.

At present, there is no MRI-related literature on radiation injury of the thyroid. The aim of this study is to measure and analyze the changes in thyroid morphology and signal intensity in patients with nasopharyngeal carcinoma before, during and after radiotherapy on 3.0 T MRI and to explore the correlation between the changes and the dose volume of the thyroid and pituitary gland. Thus, this study could provide another valuable evaluation method for the clinical diagnosis and prevention of nasopharyngeal carcinoma.

## Materials and methods

### The patient

This retrospective study was approved by the medical ethics committee of the hospital. From April 2015 to April 2024, 32 patients (14 women and 18 men; mean age 54.7 ± 12.3 years [range 25–80 years]) who were initially diagnosed with nasopharyngeal carcinoma and received radiotherapy in our hospital were enrolled. Only patients without thyroid, pituitary and hypothalamic diseases confirmed by pathology were included. IMRT was used to complete the intended radiotherapy in all patients. All patients underwent MRI with T1-weighted, T2-weighted, and contrast-enhanced T1-weighted imaging before, during, and 6 months and 12 months after radiotherapy.

### Radiotherapy methods and radiation dose volume measurement

**Figure 1:**
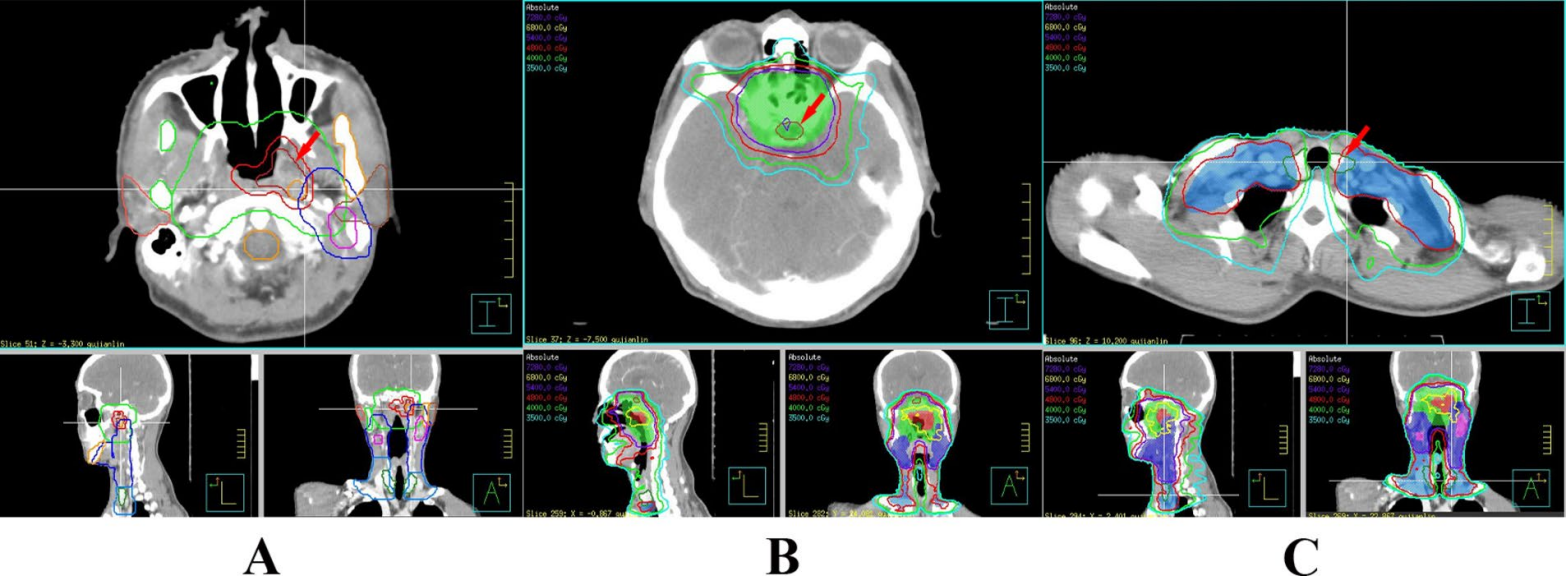
Delineation of the patient’s primary tumor **(A)**, pituitary gland **(B)**, and thyroid gland **(C)**.

**Figure 2:**
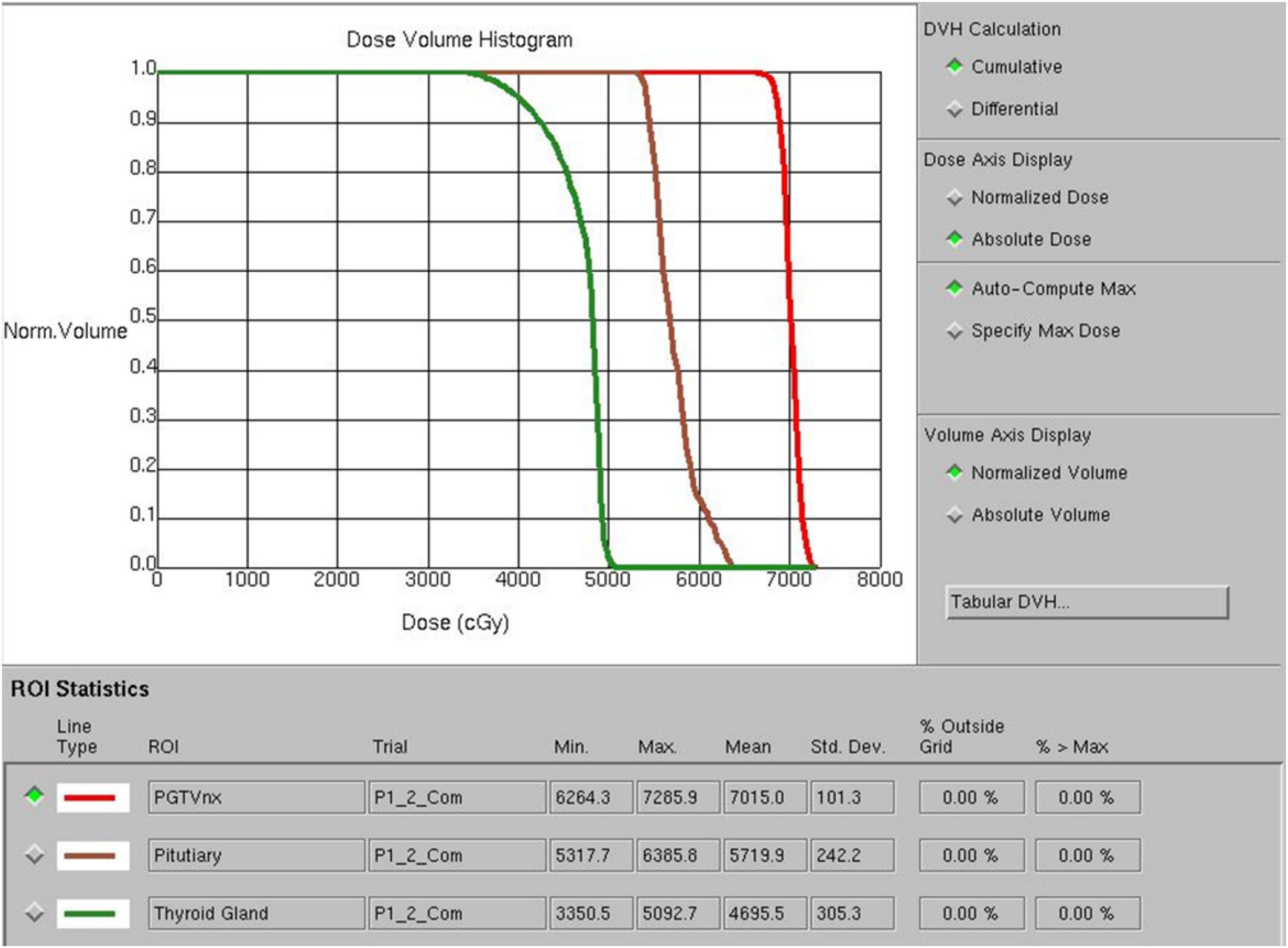
Dose volume histogram (DVH) diagram of radiotherapy.

Doctors used a treatment planning system (TPS) to design radiotherapy plans, which were established according to the specific condition of patients. Patients received 6 MV X-ray or electron linear accelerator general radiotherapy five times a week, using 200 cGy each time, with a neck cure dose of DT 65–75 Gy and a prevention dose of DT 55–60 Gy. The slice distance and slice thickness of the positioning images were all 3 mm. After scanning, the images were sent to the treatment planning system, and the thyroid contour and other target areas were delineated layer by layer (Figure 1). The dose volume histogram (DVH) was used to calculate the dosimetric parameters of thyroid and pituitary irradiation, including the maximum dose, minimum dose, and average dose of the thyroid and pituitary, and the Vx of the thyroid (the percentage of the thyroid volume irradiated by x Gy dose, *x* = 30, 35, 40, 45, 50) (Figure 2).

### MRI technique

A Philips Achieva 3.0 T superconducting MR machine and a head and neck combined phased array coil were used. The scanning scope included the whole thyroid gland. T1WI and enhanced T1WI scans were performed with a spin echo sequence (SE) (TR 650–710 ms, TE 15 ms, FOV 180 mm, matrix 256 × 512, excitation times 1, slice thickness 6 mm, interval 1 mm). Contrast-enhanced scanning was performed with Gd-DTPA injection through a high-pressure autoinjector (XD 2000 CT/MRI, Ulrich GMBH, Ulrich, Germany, Germany) by bolus injection into the forearm vein at a rate of 2 mL/s at a dose of 0.1 mmol/kg. T2WI scans were performed with a turbo echo sequence (TSE) (TR 2800–4,900 ms, TE 80–120 ms, FOV 180 mm, matrix 256 × 512, excitation times 1 time, slice thickness 6 mm, interval 1 mm). The scanning parameters were consistent before and after radiotherapy.

### Analysis of images

Philips Extended MR WorkSpace software was used to measure the following data. The transverse and anteroposterior diameters were measured on the axial T1WI images. Then, the outline of the thyroid gland was manually drawn as the region of interest (ROI) on each layer of the image, and the thyroid volume was calculated automatically by software. The percentage of thyroid volume (PTV) before and after radiotherapy was calculated [PTV = (postradiotherapy thyroid volume/preradiotherapy thyroid volume) × 100%]. More than 100% was considered an increase, and less than 100% was considered a decrease. The signal intensity (SI) of the bilateral lobe of the thyroid and surrounding fat on T1WI, T2WI, and enhanced T1WI axial images was measured by drawing the ROI. The SI of both lobes was averaged and used as the mean SI of the thyroid gland. The relative signal intensity (RSI) of the thyroid in each sequence scan was calculated by the formula:

T1WI RSI = T1WI SI thyroid/T1WI SI fat.

T2WI RSI = T2WI SI thyroid/T2WI SI fat.

Enhanced T1WI RSI = enhanced T1WI SI thyroid/T1WI SI fat.

## Results

The demographic and clinical characteristics of the patients are listed in Table 1. Table 2 shows the dose to the thyroid gland and the dose to the pituitary gland.

**Table 1:** Demographic and clinical characteristics of the patients.

Characteristic	Number of patients
Age (years)	
≥60	12
<60	20
Gender	
Male	18
Female	14
Poorly differentiated squamous cell carcinoma	5
Undifferentiated non-keratinizing carcinoma	27
T stage	
T1	2
T2	14
T3	9
T4	7
N stage	
N0	3
N1	7
N2	21
N3	1
Clinical stage	
I	2
II	5
III	18
IV	7

**Table 2:** Radiation dose of the patients (Mean ± SD).

Indicators	Statistics
Thyroid dose (cGy)	
Maximum (Dmax)	6449.9 ± 493.1
Minimum (Dmin)	2930.9 ± 1058.0
Mean (Dmean)	4794.2 ± 1027.7
V30	89.44 ± 24.04%
V35	85.77 ± 24.60%
V40	79.95 ± 24.69%
V45	72.11 ± 24.36%
V50	55.71 ± 25.65%
Pituitary dose (cGy)	
Maximum	5934.6 ± 894.8
Minimum	4567.1 ± 1054.5
Mean	5270.2 ± 902.7

### Morphology

**Figure 3:**
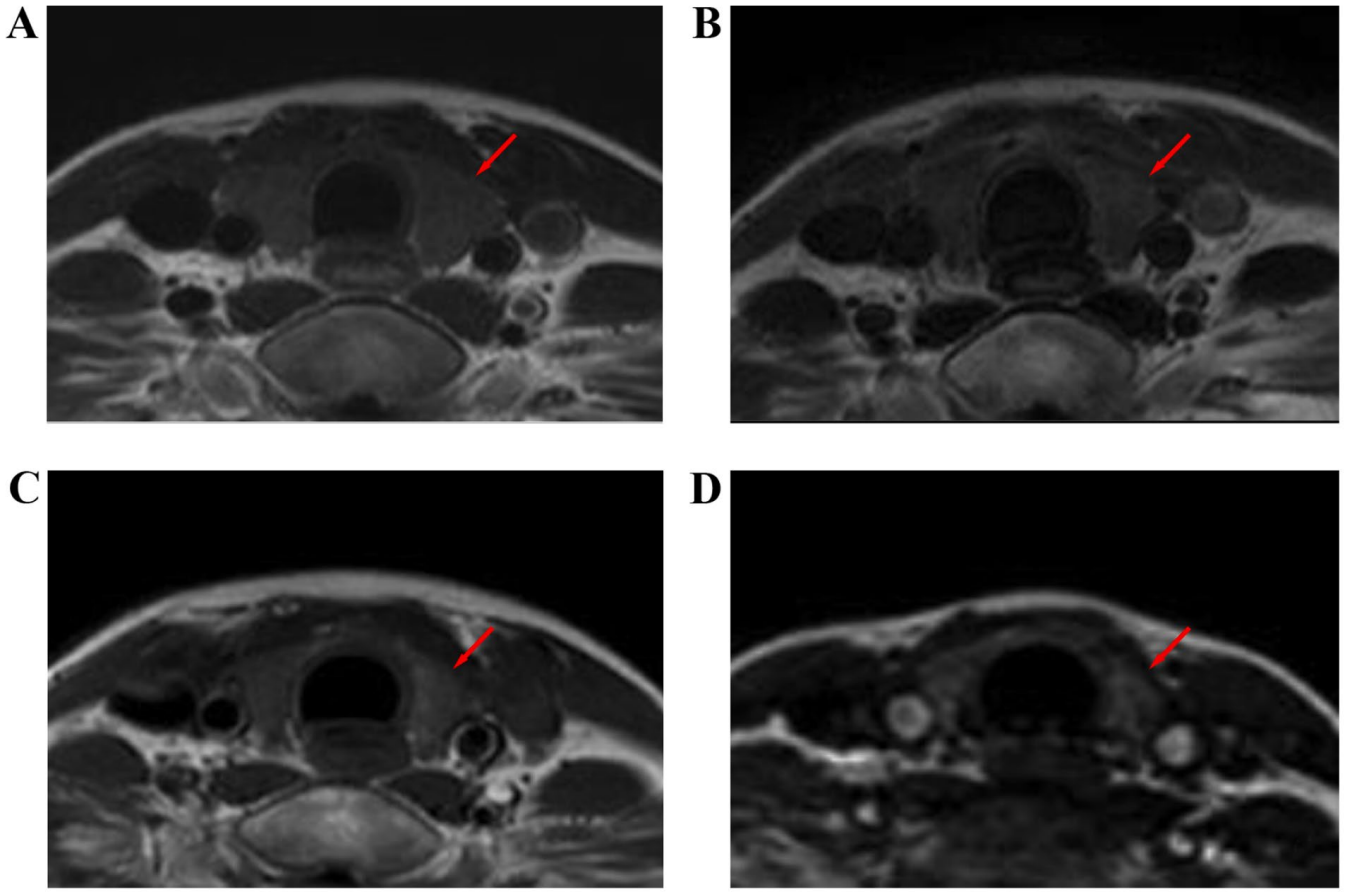
Thyroid size at different time points before and after radiotherapy for nasopharyngeal carcinoma: T1WI images before radiotherapy **(A)**, during radiotherapy **(B)**, 6 months after radiotherapy **(C)**, and 12 months after radiotherapy **(D)**.

The transverse and anteroposterior diameters of each lobe and the volume of the thyroid gland gradually decreased from those before radiotherapy to those during radiotherapy and those 6 months and 12 months after radiotherapy (Table 3 and Figure 3). The PTV during radiotherapy, 6 months after radiotherapy and 12 months after radiotherapy were (87.34 ± 8.90)%, (66.47 ± 8.68)% and (50.87 ± 8.39)% of those before radiotherapy, respectively, and these values were negatively correlated with the mean dose and V50 of the thyroid (*p* < 0.05). There was a significant negative correlation between PTV and V40 and V45 of the thyroid during and 12 months after radiotherapy. There was a weak negative correlation between PTV and V35 of the thyroid at 12 months after radiotherapy. There was no significant correlation between PTV and V45, V40 or V35 at 6 months after radiotherapy. There was no significant correlation between PTV and V35 during radiotherapy. There was no significant correlation between PTV and the maximum radiation dose, the minimum radiation dose and the V30 of the thyroid and the irradiation dose of the pituitary at each time point after radiotherapy (*p* > 0.05) (Table 4).

**Table 3:** Thyroid diameter and volume at different time points before and after radiotherapy (mean ± SD).

Thyroid gland	Before radiotherapy	During radiotherapy	After radiation therapy		*F*	*P*
			6 months	12 months		
Transverse diameter of right lobe (mm)	15.42 ± 2.34	14.67 ± 2.28	13.35 ± 2.10	12.11 ± 2.16	13.845	<0.001
Anteroposterior diameter of right lobe (mm)	15.14 ± 2.69	13.81 ± 2.33	12.98 ± 2.34	11.79 ± 2.27	11.625	<0.001
Transverse diameter of left lobe (mm)	18.09 ± 3.20	17.19 ± 2.81	15.26 ± 2.80	13.81 ± 2.23	15.254	<0.001
Anteroposterior diameter of left lobe (mm)	16.27 ± 2.80	15.42 ± 2.50	14.07 ± 2.46	12.89 ± 2.12	11.517	<0.001
Volume (cm^3^)	19.64 ± 4.78	17.07 ± 4.78	13.06 ± 3.52	9.97 ± 3.01	37.188	<0.001

**Table 4:** Correlation between percentage of thyroid volume and radiation dose.

Indicators	PVT during radiotherapy		PVT after radiotherapy			
			6 months		12 months	
	*r*	*p*	*r*	*p*	*r*	*p*
Thyroid gland dose						
Mean	−0.515	0.003	−0.426	0.015	−0.501	0.003
Maximum	−0.001	0.995	−0.339	0.057	−0.103	0.575
Minimum	−0.146	0.424	−0.044	0.812	−0.306	0.089
V30	−0.267	0.139	−0.166	0.364	−0.234	0.154
V35	−0.350	0.052	−0.203	0.266	−0.390	0.027
V40	−0.457	0.009	−0.298	0.097	−0.474	0.006
V45	−0.555	0.001	−0.329	0.066	−0.495	0.004
V50	−0.673	0.000	−0.358	0.044	−0.481	0.005
Pituitary radiation dose						
Mean	−0.180	0.325	−0.178	0.330	−0.122	0.508
Maximum	−0.113	0.539	−0.068	0.771	−0.141	0.441
Minimum	−0.174	0.341	−0.206	0.258	−0.106	0.563

### Relative signal intensity

**Figure 4:**
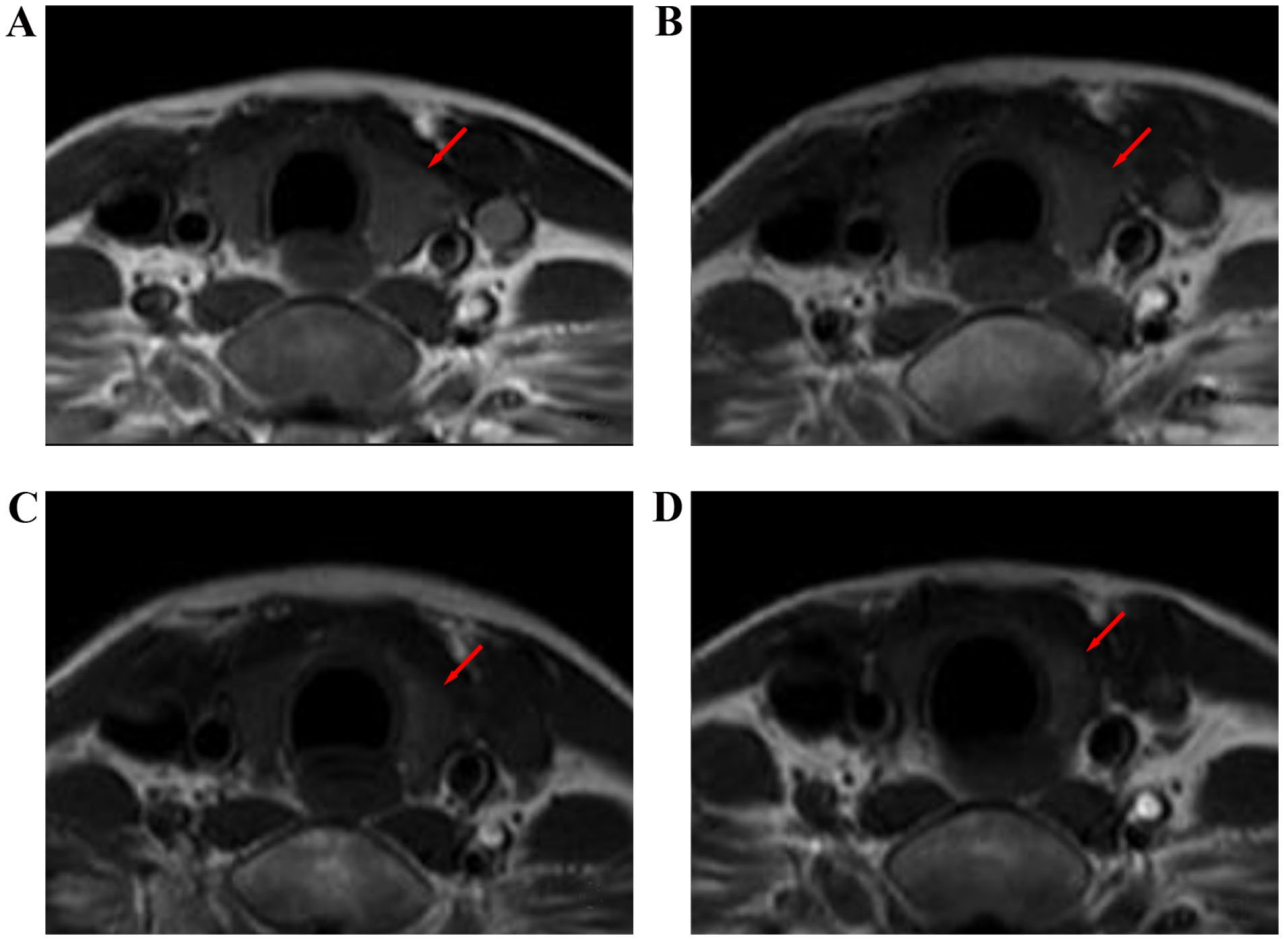
T1-weighted images of the thyroid gland of a 32-year-old female with nasopharyngeal carcinoma before and after radiotherapy. **(A)** Before radiotherapy, **(B)** during radiotherapy, **(C)** 6 months after radiotherapy, and **(D)** 12 months after radiotherapy.

**Figure 5:**
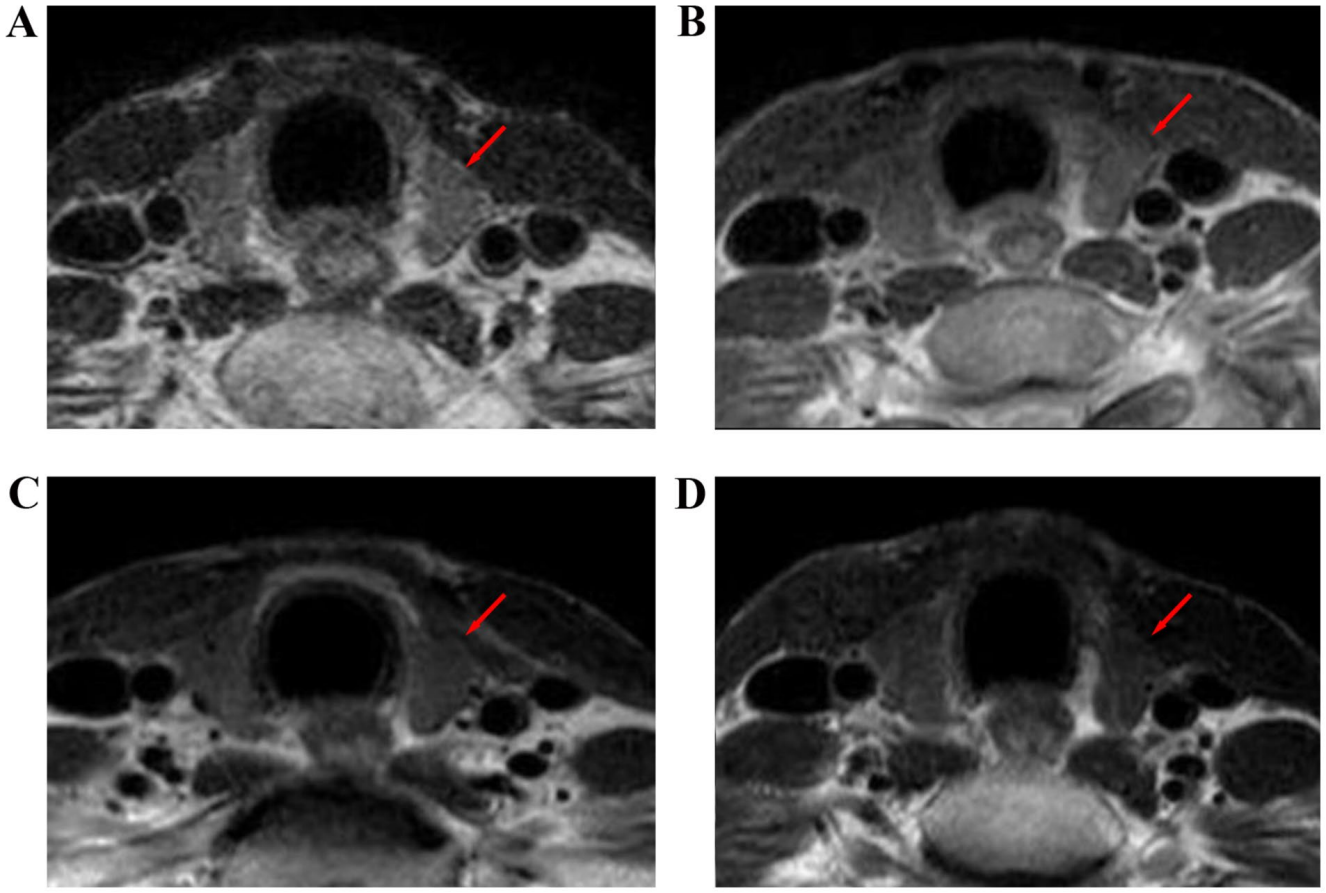
T2-weighted images of the thyroid gland before and after radiotherapy for nasopharyngeal carcinoma. **(A)** Before radiotherapy, **(B)** during radiotherapy, **(C)** 6 months after radiotherapy, and **(D)** 12 months after radiotherapy.

**Figure 6:**
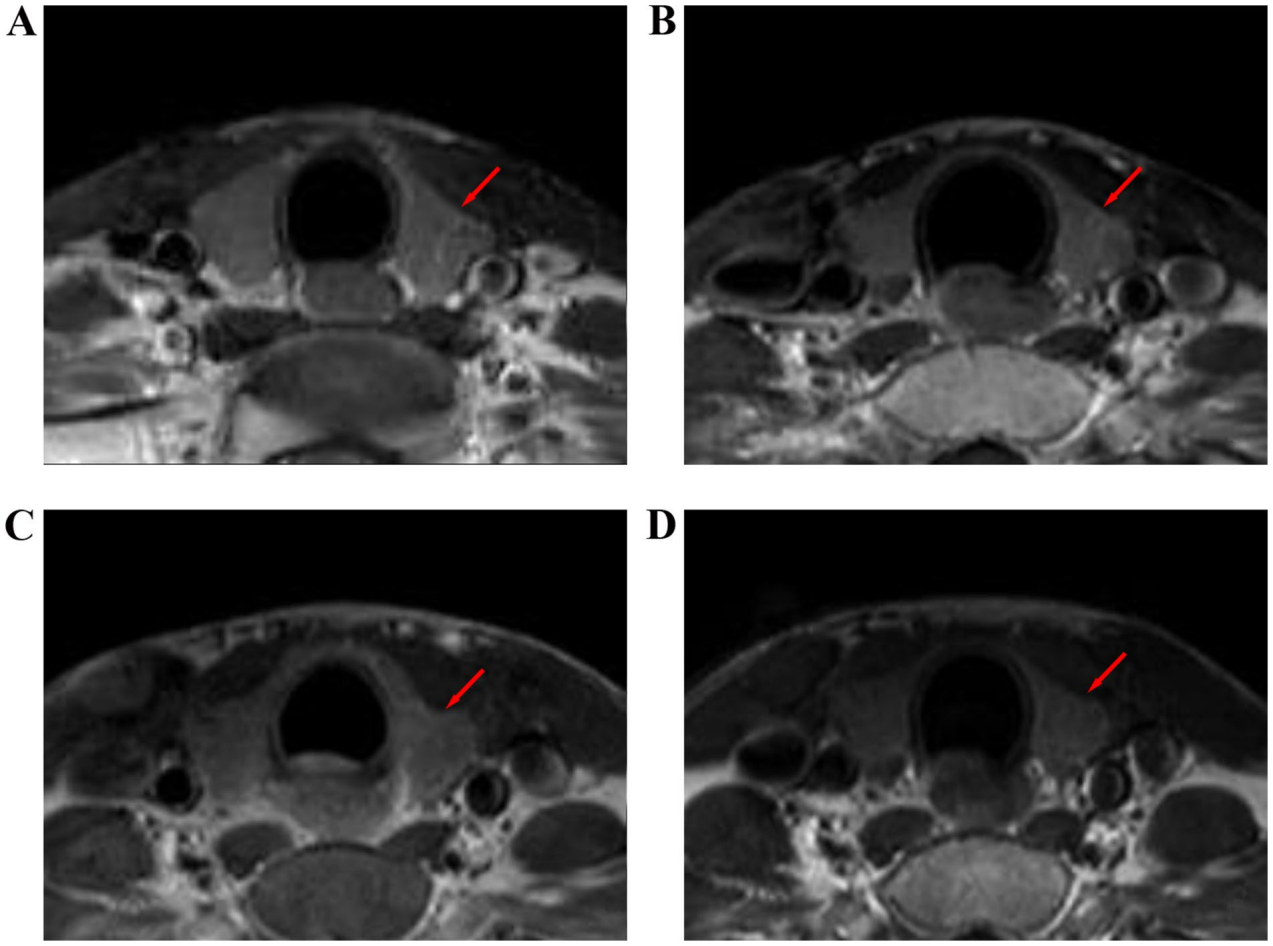
Contrast enhanced T1WI images of the thyroid gland before and after radiotherapy for nasopharyngeal carcinoma. **(A)** Before radiotherapy, **(B)** during radiotherapy, **(C)** 6 months after radiotherapy, and **(D)** 12 months after radiotherapy.

The mean T1WI RSI, T2WI RSI, and contrast-enhanced T1WI RSI of the thyroid gland gradually decreased from before radiotherapy to during radiotherapy and at 6 months and 12 months after radiotherapy (Table 5 and Figures 4–6). Tables 6–8 show the correlation between the MRI signal intensity of the thyroid after radiotherapy and the irradiated dose volume. The T1WI RSI, T2WI RSI and enhanced T1WI RSI of the thyroid during radiotherapy, 6 months and 12 months after radiotherapy were negatively correlated with the mean radiation dose, V50, V30, V35, V40, and V45 of the thyroid, respectively (*p* < 0.05), but were not significantly correlated with the maximum radiation dose, minimum radiation dose of the thyroid gland and the radiation dose of the pituitary gland (*p* > 0.05).

**Table 5:** MRI parameters of thyroid at different time points before and after radiotherapy (mean ± SD).

Thyroid gland	Before radiotherapy	During radiotherapy	After radiotherapy	
			6 months	12 months
T1WI RSI	0.398 ± 0.076	0.370 ± 0.065	0.330 ± 0.060	0.301 ± 0.061
T2WI RSI	0.413 ± 0.076	0.381 ± 0.068	0.335 ± 0.066	0.303 ± 0.064
Enhanced T1WI RSI	0.675 ± 0.154	0.620 ± 0.139	0.523 ± 0.124	0.489 ± 0.120

**Table 6:** Correlation between thyroid T1WI RSI and radiation dose.

Indicators	T1 during radiotherapy		T1 after radiotherapy			
			6 months		12 months	
	*r*	*p*	*r*	*p*	*r*	*p*
Thyroid gland dose						
Mean	−0.436	0.000	−0.453	0.000	−0.466	0.000
Maximum	−0.133	0.296	−0.034	0.791	−0.107	0.402
Minimum	−0.205	0.105	−0.117	0.362	−0.155	0.220
V30	−0.329	0.008	−0.324	0.009	−0.365	0.003
V35	−0.389	0.002	−0.399	0.001	−0.428	0.000
V40	−0.455	0.000	−0.460	0.000	−0.473	0.000
V45	−0.511	0.000	−0.510	0.000	−0.515	0.000
V50	−0.520	0.000	−0.546	0.000	−0.536	0.000
Pituitary radiation dose						
Mean	−0.022	0.864	−0.050	0.695	−0.001	0.993
Maximum	−0.027	0.832	−0.088	0.491	−0.034	0.792
Minimum	−0.018	0.885	−0.038	0.763	−0.014	0.911

**Table 7:** Correlation between thyroid T2WI RSI and radiation dose.

Indicators	T2 during radiotherapy		T2 after radiotherapy			
			6 months		12 months	
	*r*	*p*	*r*	*p*	*r*	*p*
Thyroid gland dose						
Mean	−0.453	0.000	−0.345	0.005	−0.380	0.023
Maximum	−0.146	0.251	−0.116	0.360	−0.070	0.582
Minimum	−0.163	0.197	−0.096	0.452	−0.156	0.217
V30	−0.363	0.003	−0.252	0.038	−0.247	0.049
V35	−0.434	0.000	−0.296	0.017	−0.324	0.009
V40	−0.483	0.000	−0.371	0.003	−0.386	0.002
V45	−0.541	0.000	−0.442	0.000	−0.449	0.000
V50	−0.614	0.000	−0.579	0.000	−0.594	0.000
Pituitary radiation dose						
Mean	−0.129	0.311	−0.228	0.075	−0.217	0.085
Maximum	−0.127	0.318	−0.203	0.108	−0.210	0.097
Minimum	−0.076	0.553	−0.215	0.088	−0.212	0.093

**Table 8:** Correlation between thyroid enhanced T1WI RSI and radiation dose.

Indicators	Enhanced T1WI during radiotherapy		Enhanced T1WI after radiotherapy			
			6 months		12 months	
	*r*	*p*	*r*	*p*	*r*	*p*
Thyroid gland dose						
Mean	−0.374	0.002	−0.356	0.004	−0.380	0.002
Maximum	−0.021	0.870	−0.064	0.616	−0.007	0.956
Minimum	−0.201	0.111	−0.169	0.183	−0.203	0.107
V30	−0.275	0.028	−0. 251	0.046	−0.289	0.020
V35	−0.285	0.023	−0.255	0.042	−0.298	0.017
V40	−0.311	0.012	−0.283	0.023	−0.319	0.010
V45	−0.349	0.005	−0.319	0.010	−0.351	0.004
V50	−0.400	0.001	−0.351	0.004	−0.376	0.002
Pituitary radiation dose						
Mean	−0.023	0.856	−0.031	0.809	−0.035	0.781
Maximum	−0.058	0.647	−0.040	0.756	−0.057	0.655
Minimum	−0.001	0.994	−0.016	0.899	−0.014	0.912

## Discussion

Both X-ray radiotherapy and electron radiotherapy are the main methods of radiotherapy. Although the electron radiotherapy dose distribution is more concentrated, but it has a clear “rapidly decreasing dose” region, so electron radiotherapy is generally used for superficial tumors. X-ray radiotherapy is applied to deep tumors and it is the therapy method for nasopharyngeal carcinoma in clinical. Radiotherapy can cause different degrees of thyroid damage in patients with nasopharyngeal carcinoma, which may cause various histopathological changes in the thyroid. Thyroid complications, such as hypothyroidism, hyperthyroidism, benign adenoma, Graves’ disease and even thyroid cancer, have been reported in the literature; among these complications, hypothyroidism is the most common one (7, 10, 11).

In this study, the thyroid volume gradually decreased with time after radiotherapy. Previous studies have found that the cause of radiation-induced thyroid injury is mainly related to vascular injury, parenchymal cell injury and autoimmune response (12–14). After radiation, thyroid cells undergo DNA strand damage in acute changes, which leads to mitotic death of thyroid cells (15). Damaged cells can still maintain their basic morphology and function, and due to the slow renewal of thyroid tissue cells, damaged cells can continue to exist and function for a long time. Therefore, radiation-induced thyroid injury often manifests gradually after a long time. The chronic changes occurred mainly months to years after radiotherapy and were characterized by massive death of thyroid cells and inhibition of thyroid cell replication by proliferating fibers (16). In addition, the products of radiation damage may increase thyroid autoantibodies (17, 18) and promote the occurrence of thyroid autoimmunity.

In terms of the rate of decrease, the decrease in thyroid volume was most obvious at 6 months after radiotherapy. The reason for this is that there is acute inflammatory edema of the thyroid gland and the compensatory effect of the thyroid gland during radiotherapy. The fibrous tissue and other tissues increase in the late stage (more than 1 year) after radiotherapy (19). However, the early stage (less than 6 months) after radiotherapy is mainly dominated by cell death (20), so the volume decrease is the fastest.

The thyroid-enhanced T1WI RSI gradually decreased with time, which was considered to be mainly related to delayed vascular damage after radiotherapy. On the one hand, the structure and function of vascular smooth muscle cells and vascular endothelial cells are changed, cell apoptosis is increased, and cell proliferation is inhibited after radiotherapy. On the other hand, various inflammatory mediators and cytokines produced in the irradiated area can mediate the local inflammatory response, and the two interact together to lead to vascular injury (21, 22). Vascular injury can occur in all levels of arteries and veins, but it most commonly occurs in microvessels, especially capillaries and venous sinuses (23, 24). Such injuries lead to vascular fibrosis, vascular diameter reduction, occlusion, and blood supply reduction and then cause thyroid injury. Enhanced T1WI RSI increased in some patients after radiotherapy. The reason for this was considered to be the decrease in thyroid hormone in blood, the increase in thyroid stimulating hormone (TSH) secretion by feedback stimulation, and the increase in thyroid blood flow.

The results of this study showed that there was a significant negative correlation between the mean radiation dose of the thyroid and the volume of bilateral thyroid lobes, including the transverse diameters, anteroposterior diameters and the degree of volume reduction. Colevas et al. (25) suggested that thyroid dysfunction could occur after exposure to more than 30–45 Gy of radiation. Zhai et al. (26) showed a significant correlation between the radiation dose to the thyroid and the incidence of hypothyroidism. The mean radiation dose to the thyroid gland in our study was higher than the above dose, and this study confirmed the above findings from a certain angle. In this study, the transverse diameter, anteroposterior diameter and volume reduction of the thyroid gland were negatively correlated with the V40, V45, and V50 of the thyroid gland. The correlation with V50 was the most significant, which was similar to the results of previous study (27). Alterio et al. (28) also found that V50 was one of the most important predictors of hypothyroidism after radiotherapy by LASSO analysis. One adult-based study (29) stated that volume reductions usually occurred prior to hypothyroidism, which could imply that thyroid hormone substitution therapy should be started early.

The T1WI RSI of the thyroid gland at 12 months after radiotherapy had the highest correlation with the mean radiation dose, V40, V45, and V50 of the thyroid gland, considering the gradual aggravation of tissue fibrosis in radiation-induced thyroid injury with time after radiotherapy. The correlation between T2WI RSI and mean radiation dose, V40, V45, and V50 of the thyroid gland was the highest during radiotherapy, considering the existence of tissue edema at this time. The correlation between enhanced T1WI RSI and mean radiation dose, V40, V45, and V50 of the thyroid gland was higher during radiotherapy and at 12 months after radiotherapy than at 6 months after radiotherapy because the inflammatory edema of the former was the most severe, and the vascular injury of the latter was the most severe. An ultrasound study found a significant increase in all color Doppler ultrasonography (CDU) parameters of the inferior thyroid artery after early radiotherapy. This may indicate that radiation-induced acute thyroiditis, which can lead to subsequent hypothyroidism or hyperthyroidism, is the main damage to the thyroid (30).

Radiation damage to the pituitary and thalamus after radiotherapy can cause central hypothyroidism. It has been reported that radiotherapy can directly inhibit the normal function of the anterior pituitary, leading to a decrease in the secretion of TSH. According to Graffeo et al. (31), when the radiation dose of the pituitary gland exceeds 45 Gy, pituitary dysfunction will begin to appear. Pomeraniec et al. (32) showed that central hypothyroidism has a long latency and may be a late side effect. Darzy and Shalet (33) showed that the pituitary dose threshold can reach 60 Gy, and hypothyroidism generally occurs 2–5 years after radiotherapy. In this study, the thalamus was not exposed to the radiation field. The mean radiation dose to the pituitary gland was 52 Gy, which was within the range of the pituitary control limit of 50–55 Gy. The follow-up time was relatively short, and no obvious pituitary injury was generally observed. There was no significant correlation between these parameters and the radiation dose to the pituitary gland. It is suggested that the changes in thyroid volume and signal intensity after radiotherapy are not related to central hypothyroidism.

## Conclusion

Current studies have shown that head and neck radiotherapy can cause hypothyroidism in patients with nasopharyngeal carcinoma, which is characterized by reduced thyroid volume and decreased signal intensity. Magnetic resonance imaging can provide useful additional information in patients with nasopharyngeal carcinoma undergoing radiotherapy.

## Data Availability

The raw data supporting the conclusions of this article will be made available by the authors, without undue reservation.
